# Down-Regulation of Decapping Protein 2 Mediates Chronic Nicotine Exposure-Induced Locomotor Hyperactivity in *Drosophila*

**DOI:** 10.1371/journal.pone.0052521

**Published:** 2012-12-26

**Authors:** Jing Ren, Jinghan Sun, Yunpeng Zhang, Tong Liu, Qingzhong Ren, Yan Li, Aike Guo

**Affiliations:** 1 State Key Laboratory of Neuroscience, Institute of Neuroscience, Shanghai Institutes for Biological Sciences, Chinese Academy of Sciences, Shanghai, China; 2 State Key Laboratory of Brain and Cognitive Science, Institute of Biophysics, Chinese Academy of Sciences, Beijing, China; Wake Forest University, United States of America

## Abstract

Long-term tobacco use causes nicotine dependence via the regulation of a wide range of genes and is accompanied by various health problems. Studies in mammalian systems have revealed some key factors involved in the effects of nicotine, including nicotinic acetylcholine receptors (nAChRs), dopamine and other neurotransmitters. Nevertheless, the signaling pathways that link nicotine-induced molecular and behavioral modifications remain elusive. Utilizing a chronic nicotine administration paradigm, we found that adult male fruit flies exhibited locomotor hyperactivity after three consecutive days of nicotine exposure, while nicotine-naive flies did not. Strikingly, this chronic nicotine-induced locomotor hyperactivity (cNILH) was abolished in *Decapping Protein 2* or *1* (*Dcp2* or *Dcp1*) -deficient flies, while only *Dcp2-*deficient flies exhibited higher basal levels of locomotor activity than controls. These results indicate that Dcp2 plays a critical role in the response to chronic nicotine exposure. Moreover, the messenger RNA (mRNA) level of *Dcp2* in the fly head was suppressed by chronic nicotine treatment, and up-regulation of *Dcp2* expression in the nervous system blocked cNILH. These results indicate that down-regulation of *Dcp2* mediates chronic nicotine-exposure-induced locomotor hyperactivity in *Drosophila*. The decapping proteins play a major role in mRNA degradation; however, their function in the nervous system has rarely been investigated. Our findings reveal a significant role for the mRNA decapping pathway in developing locomotor hyperactivity in response to chronic nicotine exposure and identify *Dcp2* as a potential candidate for future research on nicotine dependence.

## Introduction

Nicotine, the major compound responsible for tobacco dependence, causes more than five million deaths worldwide every year and has been strongly implicated in various neuropsychiatric disorders [1]. A single exposure to nicotine induces both immediate and long-lasting responses [2], [3], while repeated or long-term nicotine exposure leads to more complicated responses at the molecular and behavioral levels, and the latter may ultimately lead to nicotine dependence [4]. It is recognized that nicotinic acetylcholine receptors (nAChRs) and the cAMP/CREB signal pathway are required for mediating the effects of nicotine [5], [6] and that the expression level of nAChRs is regulated by multiple exposures to nicotine [7], [8], [9]. High-throughput work indicates that the expression of numerous molecules changes upon nicotine exposure [10], [11], [12]; however, only a few of the identified molecules have been validated. In addition, the overall profile of the gene regulation and the behavioral changes that are induced by nicotine exposure, especially by long-term nicotine exposure, remain unclear.

Invertebrate animal models, like *Caenorhabditis elegans* and *Drosophila melanogaster*, have been used to study the effects of addictive drugs, such as cocaine, ethanol, and nicotine [13], [14], [15], [16]. Although research on the effects of nicotine in fruit flies began fifty years ago, the work has mostly focused on nicotine resistance, as nicotine can be used as an insecticide [17]. In the past decade, it has been reported that fruit flies display rapid onset hyperactivity and spasmodic movement when exposed to volatilized nicotine; in addition, similar to mammals, dopaminergic signaling and the cAMP/CREB pathway play important roles in these effects [18], [19], [20], [21], indicating that some conserved mechanisms are shared between the fruit fly and mammals.

Locomotor hyperactivity is an obvious symptom of many neuropsychiatric disorders, such as mania and attention-deficit hyperactivity disorder (ADHD) [22], [23], and is a simple parameter that can be used to measure drug effects [24], [25]. In rats, acute and chronic nicotine exposure causes changes in locomotion, including locomotor hyperactivity [26]. In this work, we used a chronic nicotine administration paradigm in *Drosophila* and found that flies exhibited locomotor hyperactivity after a few days of nicotine treatment. We further identified Dcp2 as a key molecule in the mediation of this chronic nicotine-induced effect.

Dcp2 is a member of the Nudix family of pyrophosphatases and was originally identified a decade ago through a yeast genetic screen [27]. Parallel studies have shown that Dcp2 is one of the major components of the decapping complex and is conserved in worms, flies, plants, mice, and humans [28]. Dcp1 is an important activator of Dcp2, and they function together as a holoenzyme to cleave the 5′ cap structure of mRNA [29], [30], [31]. The decapping signal plays an important function in mRNA turnover and translation, which widely affects gene expression [32], [33], [34]. In addition, Dcp1 and some P-body factors that are involved in the control of decapping have been found to be expressed in neurons and have been suggested to affect synaptic plasticity-related modifications of neural activity in *Drosophila* [35], [36]. However, there is little known regarding Dcp2 function in the nervous system or how Dcp2 and Dcp1 expression is regulated by internal signals or external stimuli. Our findings indicate that chronic nicotine administration regulates Dcp2 expression and that decapping signaling may play an important role in mediating nicotine-induced effects. These results provide a foundation for future research on the molecular mechanisms of the complex behavioral changes that are induced by chronic nicotine exposure.

## Results and Discussion

### Chronic Nicotine Administration Induced Locomotor Hyperactivity in Adult Male Flies

**Figure 1:**
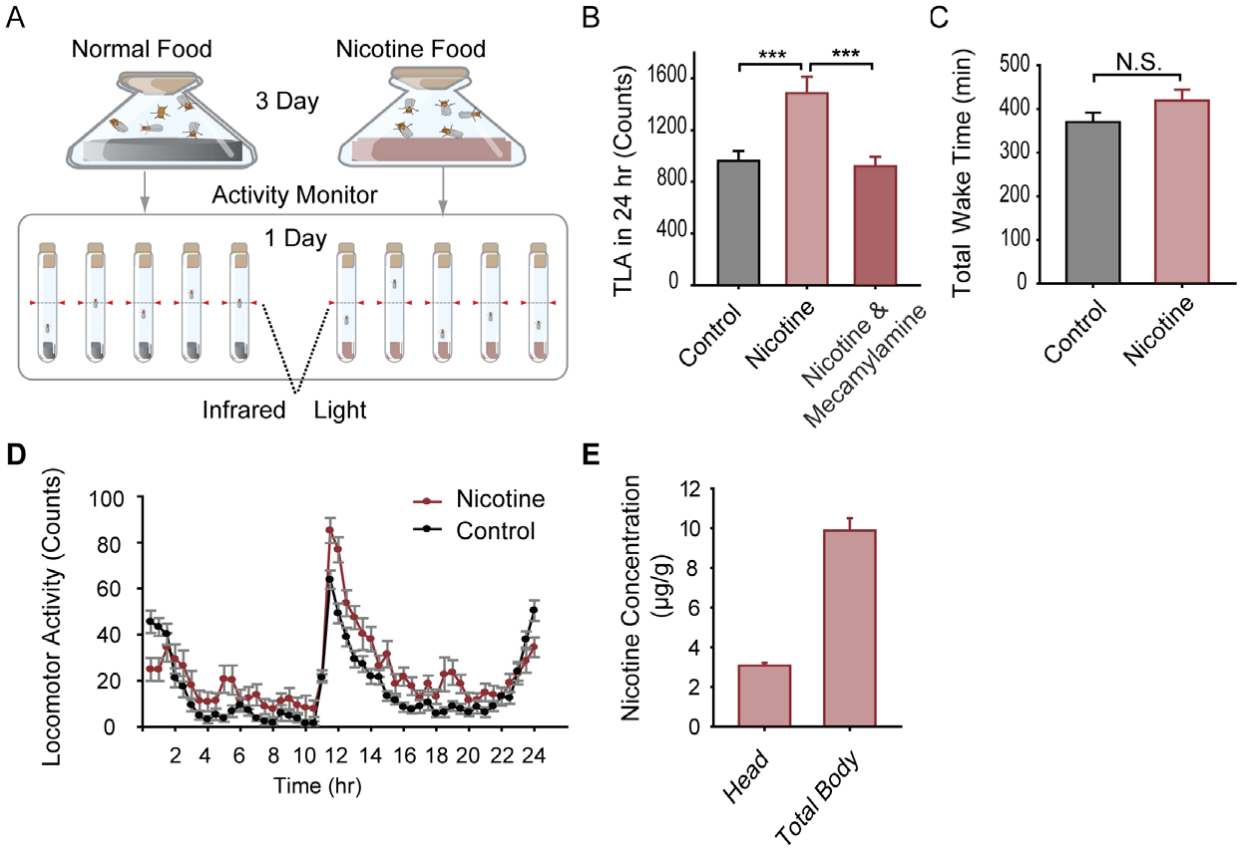
Long-term nicotine administration via food intake induces locomotor hyperactivity in wild-type CS flies. A. Schematic of the nicotine administration and locomotor activity assay. Adult male flies were fed with normal or nicotine-containing food for 3 days in vials and then transferred into individual recording tubes. Locomotor activity was monitored for 24 hr. B. Total locomotor activity (TLA) over 24 hr in CS flies treated with 3 mM nicotine was significantly higher than that of flies treated with normal food and was blocked by the addition of 0.75 mM mecamylamine to the 3 mM nicotine-containing food in the recording tubes (n = 32 for each group, ***P<0.001, *t-*test). C. Total wake time during 24 hr for flies treated with 3 mM nicotine-containing food showed no significant difference with that for flies treated with control food (n = 32 for each group, N.S. indicates no significant difference, P>0.05, *t-*test). D. Locomotor activity curves quantified every 30 min for 24 hr for flies treated with nicotine-containing and control food showed similar distributions. E. Histogram of nicotine concentration assayed by high performance liquid chromatography (HPLC) in the heads and whole bodies of nicotine-treated flies. Nicotine was not detectable in control flies treated with normal food. Three independent experiments were performed for each group. Bars and error bars represent the mean ± SEM.

To explore the effects of chronic nicotine administration, we exposed adult Canton-S (CS) wild-type male flies to free base nicotine (−/−) via food uptake for three days and then assayed their locomotor activity individually in nicotine-containing food for one day (Fig. 1A). Total locomotor activity (TLA) of flies fed with nicotine-containing food (at doses of 0.6, 1.8, and 3.0 mM) was significantly increased (locomotor hyperactivity) when compared to flies fed with normal food (Fig. 1B and S1), while there was no significant difference in total wake time between the nicotine (at 3.0 mM) and control groups (Fig. 1C). The 24-hour activity curve revealed that chronic nicotine administration did not change the distribution of the locomotor activity, but there was a general increase in activity during the day (Fig. 1D).

To test whether continuous nicotine treatments are required for the induction of the hyperactive behavioral response, we examined an additional two protocols. Flies were fed with normal food for 3 days and then switched to nicotine-containing food before recording, or flies were fed with nicotine-containing food for 3 days and then were switched to normal food before recording. Results showed that the TLA of both groups was comparable to the control group fed with normal food, indicating that continuous nicotine administrations are necessary for the promotion of locomotor hyperactivity (Fig. S1).

To examine how much nicotine is absorbed by the flies via food intake, we assayed nicotine concentration in the fly body and head using high performance liquid chromatography (HPLC). The nicotine concentration in flies fed with normal control food was below the lowest threshold (0.01 µg/g) detectable by HPLC. In flies fed with 3 mM nicotine-containing food for 4 consecutive days, the nicotine concentration was 9.89±0.52 µg/g in the entire body and 3.07±0.24 µg/g in the head (Fig. 1E). These results demonstrate that nicotine can be absorbed effectively via food uptake.

Given that nicotine tastes bitter to humans and that flies also exhibit dose-dependent aversive behavior in response to nicotine [37], we evaluated the consumption of nicotine-containing food. CS flies were starved for 12 hours and then fed with 3 mM nicotine-containing food or normal food. A water-soluble, edible blue dye was added to both foods for the quantification of food consumption [38]. Naive flies that had never been fed nicotine-containing food showed remarkably reduced consumption of the nicotine-containing food (16 µg per fly) compared to the consumption of normal food (32 µg per fly). Flies that were fed nicotine-containing food for 1–4 days gradually increased consumption (from 21 to 27 µg per fly) to approximately 66–84% of normal food intake (Fig. S2A–B). These results indicate that flies reduce consumption of 3 mM nicotine-containing food but that long-term exposure to nicotine can attenuate this effect.

As excessive food starvation can lead to hyperactivity in flies [39], we then tested whether the locomotor hyperactivity that was induced by chronic nicotine administration was due to the starvation effect. Flies treated with food containing 2.0 mM quinine, a bitter compound, exhibited a similar decrease in food consumption as that observed in response to the 3.0 mM nicotine-containing food (Fig. S2C). Using the same behavioral paradigm, we found that the locomotor activity level of these quinine-treated flies was slightly increased but was not significantly different from that of the control flies (Fig. S2D–E). Locomotor hyperactivity induced by chronic nicotine exposure was therefore not due to the side-effects of reduced food consumption, and thus can be referred to as chronic nicotine administration-induced locomotor hyperactivity (cNILH).

It is known that nicotine acts in the nervous system by activating nAChRs, and some nAChR subunits have been reported to play a critical function in nicotine dependence in mammalian systems [40]. In our experiments, the nicotine-induced locomotor hyperactivity effect was blocked when mecamylamine, a non-selective nAChR antagonist [41], [42], was administered in addition to nicotine during the treatment and/or recording periods (Fig. 1B), demonstrating that nAChRs are necessary for cNILH. In *Drosophila*, the genes for ten nAChR subunits have been identified and are mainly expressed in the central nervous system (CNS) [43]. To determine which subunits mediate the chronic nicotine effects observed in our model system, we knocked-down the expression of eight of the nAChR subunits, one at a time, using an RNA-interfering (RNAi) approach [44] in the nervous system via a pan-neuronal GAL4, *elav*-GAL4. In ten of the RNAi lines that we examined, cNILH was absent only in flies that expressed *α96Ab* ^RNAi^ (lines V1195 and V1194) or *gfa* ^RNAi^ (line V11329), suggesting that these two subunits are involved in the cNILH effect (Fig. S3).

### V1194 Flies Failed to Develop cNILH

**Figure 2:**
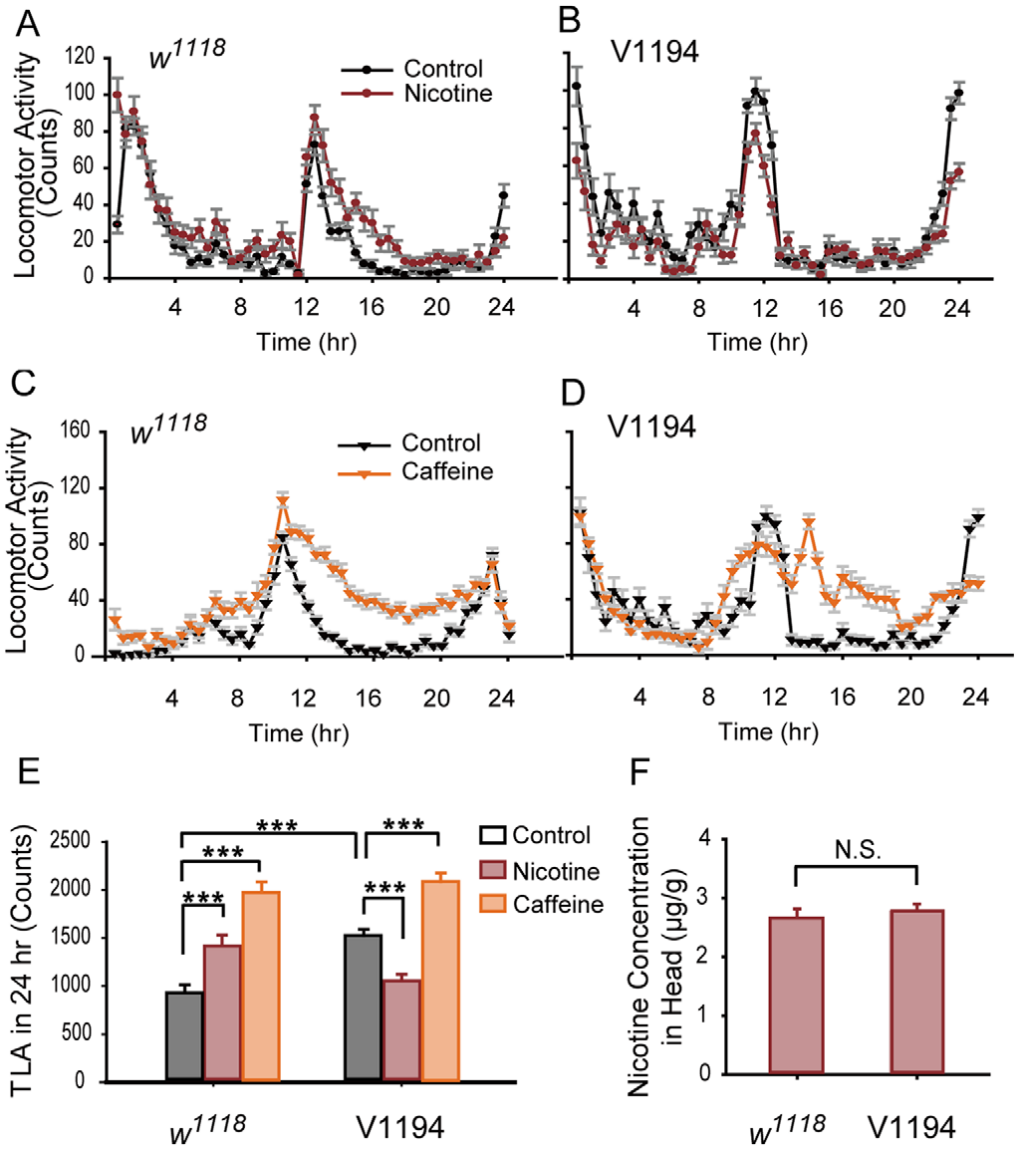
V1194 flies fail to develop chronic nicotine-exposure-induced locomotor hyperactivity. A–D. Locomotor activity was measured every 30 min for 24 hr. In A and B, *w^1118^* and V1194 flies were continuously treated with 3 mM nicotine-containing or control food for 4 days, and locomotor activity was recorded on the 4^th^ day. In C and D, *w^1118^* and V1194 flies were treated with 2.5 mg/ml caffeine-containing or control food for 1 day, and locomotor activity was recorded on the 1^st^ day. E. Total locomotor activity (TLA) during 24 hr in *w^1118^* and V1194 flies fed with control, nicotine-, or caffeine-containing food. *w^1118^* flies showed significantly higher TLA when treated with either nicotine or caffeine compared to the control group. The TLA of the V1194 flies was significantly increased when they were treated with caffeine (***P<0.001), but was decreased when treated with nicotine (***P<0.001). The basal TLA of the V1194 flies was significantly higher than the basal TLA of the *w^1118^* flies (***P<0.001). n = 28–32, two-way ANOVA. F. There was no significant difference in the nicotine concentration in the heads of the V1194 and *w^1118^* flies (three independent experiments per group, N.S. indicates no significant difference, P>0.05, *t*-test). Bars and error bars represent the mean ± SEM.

**Figure 3:**
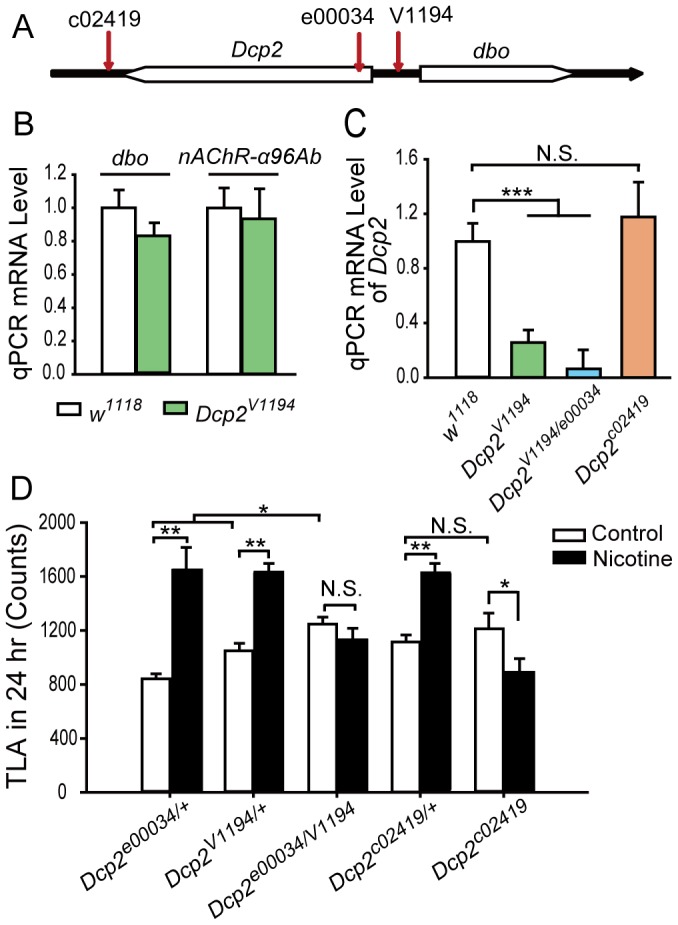
*Dcp2* mutant flies fail to develop cNILH. A. Diagram of the *Dcp2* genomic region with gene direction. The red vertical arrows indicate the P-element insertion sites of the *Dcp2^V1194^*, *Dcp2^c02419^*, and *Dcp2^e00034^* mutant alleles. B–C. Total RNA extracted from fly bodies was analyzed for *dbo, nAChR-α96Ab* and *Dcp2* mRNA levels by relative qPCR. The mRNA levels of the tested genes were normalized to *rp49* mRNA. (B) The mRNA levels of *dbo and nAChR-α96Ab* were not altered in the *Dcp2^V1194^* flies compared to the *w^1118^* flies (three independent experiments, N.S., P>0.05, *t*-test). (C) *Dcp2* mRNA levels were reduced in both *Dcp2^V1194^* and *Dcp2^V1194/e00034^* flies (***P<0.001) but not in *Dcp2^c02419^* flies (N.S., P>0.05), compared to *w^1118^* flies. Three independent experiments were performed, one-way ANOVA. D. The locomotor activity of flies treated with nicotine-containing or control food for 4 days was recorded on the 4^th^ day. Total locomotor activity (TLA) data shows cNILH in heterozygous mutant *Dcp2^V1194/+^*, *Dcp2^c02419/+^*, and *Dcp2^e00034/+^* flies (**P<0.01), but not in trans-heterozygous mutant *Dcp2^V1194/e00034^* and homozygous mutant *Dcp2^c02419^* flies (N.S., P>0.05) (Mann-Whitney U test). *Dcp2^c02419^* flies showed significantly decreased TLA (*P<0.05). Basal TLA of *Dcp2^V1194/e00034^* flies was significantly higher than that of *Dcp2^V1194/+^* or *Dcp2^e00034/+^* flies (*P<0.05), but comparable in *Dcp2^c02419^* and *Dcp2^c02419/+^* flies (N.S., P>0.05) (One-way ANOVA). n = 28–32. N.S. indicates no significant difference. Bars and error bars represent the mean ± SEM.

During our examination of nAChR subunits, we noted that locomotor hyperactivity could not be induced in V1194 flies with a UAS-*nAChR-α96Ab* ^RNAi^ insertion (Fig. 2A, B, and E). By contrast, V1195 flies with the same insertion at a different chromosomal location were able to develop hyperactivity in a manner similar to that of *w^1118^* flies, the genetic background strain of both the V1194 and V1195 lines (Fig. S4C). The mRNA level of *nAChR-α96Ab* was not altered in V1194 flies compared to *w^1118^* flies (Fig. 3B), excluding the possibility that leaky expression of *nAChR-α96Ab* ^RNAi^ disrupted the development of cNILH in V1194 flies.

We then systematically examined V1194 and *w^1118^* flies that were exposed to multiple doses of nicotine with different durations of nicotine feeding. We found that, similar to CS flies, *w^1118^* flies exposed to three doses of nicotine (1.8, 3, and 4.2 mM) exhibited significant cNILH on the 4^th^ day, whereas total locomotor activity of the V1194 flies did not increase in response to any of the nicotine doses, and even showed a significant decrease at most nicotine doses over 5 days (Figs. S4A–B). HPLC data showed that there was no significant difference in the concentration of nicotine in the head between these two fly lines (2.68±0.14 µg/g in *w^1118^* flies, 2.76±0.13 µg/g in V1194 flies, Fig. 2F). These results demonstrate that while V1194 flies have normal nicotine absorption, they have a defect in the response to chronic nicotine administration.

Of note, basal locomotor activity of V1194 flies was higher than that of *w^1118^* and V1195 flies (Fig. 2E and S4C). To test the possibility that V1194 flies cannot exhibit further increases in locomotor activity due to their already higher basal level of activity, we treated them with caffeine, which is known to induce locomotor hyperactivity after one day of treatment in flies [45]. We found that, as in *w^1118^* flies, TLA was significantly elevated in V1194 flies in response to caffeine (Fig. 2C–E). This result demonstrates that V1194 flies have the potential to exhibit higher locomotor activity, and therefore, the failure to develop cNILH is not a result of any existing locomotor defect.

### *Dcp2-*deficient Flies Fail to Develop cNILH

We then sought the true cause of the defect in V1194 flies. Using inverse PCR, we localized the insertion in the V1194 line to the 15,819,920 site of the 3L chromosome, upstream of both the *Dcp2* (CG6169) and *dbo* (CG6224) genes (Fig. 3A). Relative quantitative PCR (qPCR) results showed that in V1194 flies, *Dcp2* mRNA level was reduced to approximately 30% of that in *w^1118^* flies (Fig. 3C), whereas the *dbo* mRNA level was not significantly altered (Fig. 3B). These results narrowed down the affected gene in V1194 flies to *Dcp2*. We reasoned that V1194 flies have a mutant *Dcp2* allele, hereafter referred to as *Dcp2^V1194^*.

We then obtained two other transposon insertion-based *Dcp2* mutant fly strains, c02419 (PBac{PB}*Dcp2^c02419^*) and e00034 (PBac{RB}*Dcp2^e00034^*/TM3) [46]. The mutant allele *Dcp2^c02419^* has a *PBac*-element insertion in the 3′ untranslated region (3′UTR) of *Dcp2* and is homozygous viable, while *Dcp2^e00034^* has a *PBac*-element insertion in the 5′UTR of *Dcp2* and is homozygous lethal (Fig. 3A). To keep the genetic background of these two mutants consistent with *Dcp2^V1194^*, we outcrossed these flies to *w^1118^* flies for five generations. As mutant allele *Dcp2^e00034^* is a recessive lethal, we utilized trans-heterozygous *Dcp2^V1194/e00034^* flies in the following study. Using qPCR, we found that the mRNA level of *Dcp2* in *Dcp2^c02419^* flies was not significantly different from that of *w^1118^* flies but was dramatically reduced in *Dcp2^V1194/e00034^* flies, to a level even lower than that of *Dcp2^V1194^* flies (Fig. 3C).

Locomotor activity assays showed that both *Dcp2^c02419^* and *Dcp2^V1194/e00034^* flies failed to develop cNILH, while heterozygous flies (*Dcp2^c02419/+^*, *Dcp2^V1194/+^*, and *Dcp2^e00034/+^*) all developed cNILH (Fig. 3D). In addition, the basal activity of *Dcp2^V1194/e00034^* flies was significantly increased compared to *Dcp2^V1194/+^* and *Dcp2^e00034/+^* flies, while *Dcp2^c02419^* flies had comparable basal locomotor activity to *Dcp2^c02419^* ^/+^ flies. However, both *Dcp2^V1194/e00034^* and *Dcp2^c02419^* flies exhibited locomotor hyperactivity in response to caffeine treatment (Fig. S5), indicating that the inability to develop cNILH in these flies is not due to a potential deficiency in locomotor activity. Thus, *Dcp2* mutants with either a low *Dcp2* mRNA level (*Dcp2^V1194^ and Dcp2^V1194/e00034^*) or a deficiency in the 3′UTR regulatory region (*Dcp2^c02419^*) were incapable of developing cNILH, indicating a critical role for Dcp2 in the observed nicotine-induced effects.

### Knock-down of Dcp2 Expression in the Nervous System Increases Basal Locomotor Activity and Blocks cNILH in Flies

**Figure 4:**
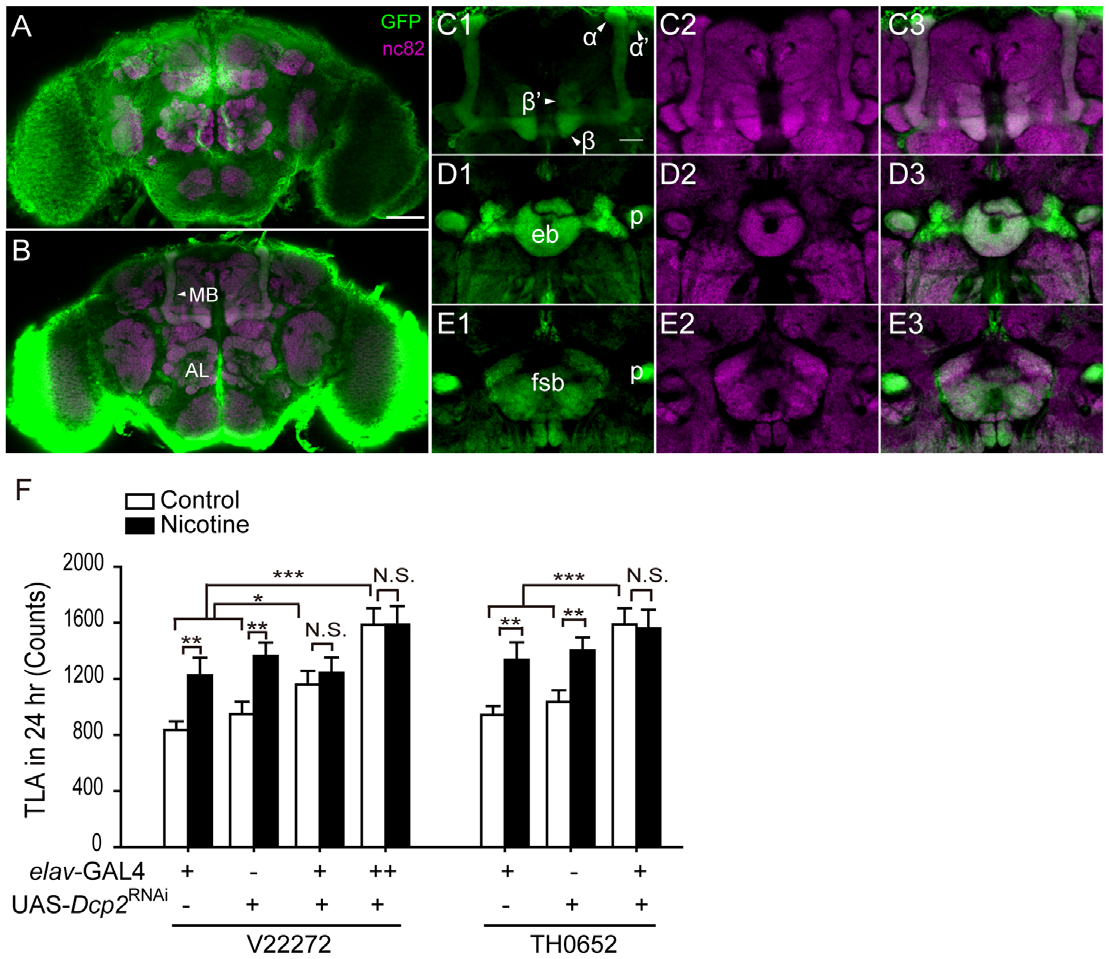
*Dcp2* knock-down in the nervous system increases basal locomotor activity and blocks cNILH. A–E. The expression pattern of *Dcp2^GAL4^* in the fly brain, visualized by mCD8::GFP. A nc82 antibody was used to visualize the neuropil. A–B. A projection view (A) and a cross-section (B) of a whole-mount brain. Scale bar represents 50 µm. MB, mushroom body; AL, antennal lobe. C–E. Dcp2 is expressed in neurons related to several major brain regions, including the α, β, α’, β’ lobes of the MB (C), the ellipsoid body (eb) (D) and the fan-shaped body (fsb) (E). Scale bar represents 20 µm. F. The total locomotor activity (TLA) of flies treated with nicotine-containing or control food for 4 days was recorded on the 4^th^ day. Two independent RNAi lines (V22272 and TH0652) were used to knock down Dcp2 in the nervous system with *elav*-GAL4. cNILH was blocked in both lines of Dcp2 knock-down flies (N.S. indicates no significant difference, P>0.05), but not in their parental control groups (**P<0.01, Mann-Whitney U test). The basal locomotor activity of both *elav*-GAL4>UAS-*Dcp2* ^RNAi−V22272^ and -*Dcp2* ^RNAi−TH0652^ flies was significantly higher than that of their parental controls and was further increased when 2 copies of *elav*-GAL4 were used to drive UAS-*Dcp2* ^RNAi−V22272^ (***P<0.001, one-way ANOVA). n = 28–32 for each group. Bars and error bars represent the mean ± SEM.

Since nicotine receptors are specifically expressed in the *Drosophila* CNS, we asked whether *Dcp2* is required in the nervous system for developing cNILH. We examined whether *Dcp2* was expressed in the fly CNS by testing the expression pattern of *Dcp2^GAL4^* (BG01766), which has a GAL4 inserted immediately downstream of the *Dcp2* promoter. We observed a ubiquitous strong signal in the fly body and head (data not shown) when visualized by a membrane-bound form of GFP (mCD8::GFP). In particular, in the adult brain, the GFP signal was widely distributed and was expressed in cell bodies on the brain surface and in the neuropil (Fig. 4A–B). Many important brain regions, such as the mushroom body, fan-shaped body, and ellipsoid body, were also labeled (Fig. 4C–E). We further validated *Dcp2* expression in tissue from the fly head using qPCR. In *elav*-GAL4>UAS-*Dcp2* ^RNAi^ flies, the *Dcp2* mRNA level was reduced to approximately 50% and 20% of that in parental controls using two RNAi strains, V22272 and TH0652, respectively (these strains have different *Dcp2* target regions; see the materials and methods section for a detailed description) (Fig. S6A). These results indicate that Dcp2 is endogenously expressed in the fly brain and can be efficiently knocked-down using an RNAi approach.

We then performed behavioral tests using *elav*-GAL4>UAS-*Dcp2* ^RNAi−V22272^ and *elav*-GAL4>UAS-*Dcp2* ^RNAi−TH0652^ flies. Both lines failed to develop cNILH, even though their parental control groups exhibited cNILH (Fig. 4F), indicating that Dcp2 function in the nervous system is critical for developing cNILH. Consistent with *Dcp2* mutant flies that had lower *Dcp2* mRNA levels (*Dcp2^V1194^* and *Dcp2^V1194/e00034^*), *elav*-GAL4>UAS-*Dcp2* ^RNAi^ flies also exhibited higher basal locomotor activity without nicotine treatment compared to their parental controls. This elevation was more obvious in *elav*-GAL4>UAS-*Dcp2* ^RNAi−TH0652^ flies with higher knock-down efficiency. The elevation was further enhanced in the *elav*-GAL4>UAS-*Dcp2* ^RNAi−V22272^ flies when two copies of *elav*-GAL4 were used (Fig. 4F). Therefore, a low level of Dcp2 in the nervous system is sufficient for the promotion of locomotor hyperactivity in flies, and chronic nicotine exposure does not further elevate the level of locomotor activity in *Dcp2-*deficient flies. It has been reported that Dcp2 shows a preference for certain mRNA targets, depending on recognition of specific sequences [47]. Thus, it is possible that deficiencies in Dcp2 expression or regulation may affect mRNA degradation unequally, possibly altering the balance of the gene expression network and thereby affecting behavioral outputs such as locomotor activity.

### Down-regulation of Dcp2 in the Nervous System Mediates cNILH

**Figure 5:**
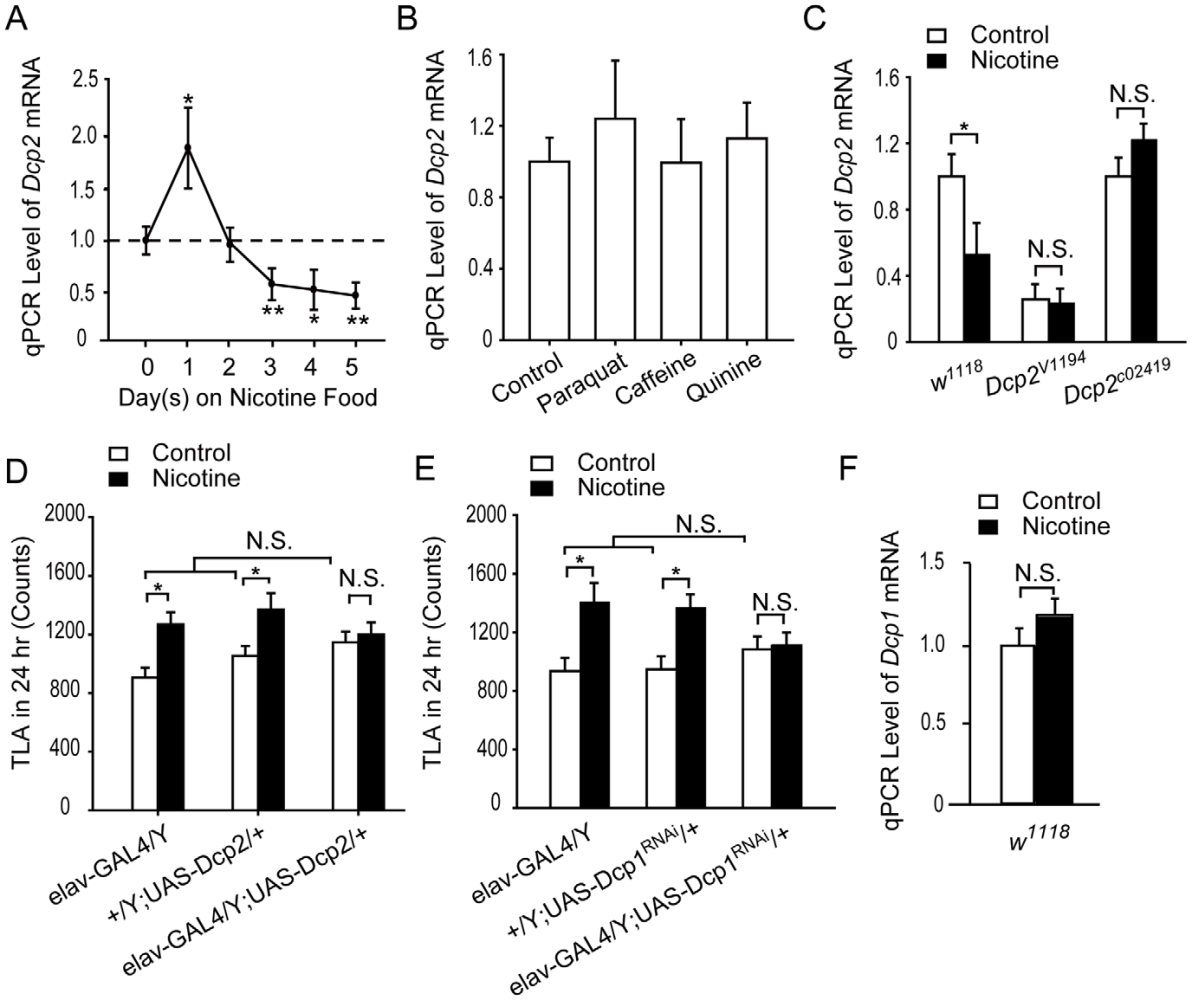
Down-regulation of *Dcp2* mediates chronic nicotine-exposure-induced locomotor hyperactivity. A–C and F. Total RNA extracted from the heads of male flies was analyzed for *Dcp2* or *Dcp1* mRNA levels by relative qPCR. mRNA levels of the tested genes were normalized to *rp49* mRNA. A. The level of *Dcp2* mRNA in *w^1118^* flies increased significantly in group 1 treated with nicotine for 1 day (*P<0.05), but decreased significantly in groups treated with nicotine for 3-, 4-, or 5-days when compared to the 0-day group without treatment (*P<0.05 in the 4-day group, **P<0.01 in the 3- and 5-day groups). Three independent experiments were performed for each group, one-way ANOVA. B. Levels of *Dcp2* mRNA was not significantly affected when *w^1118^* flies were treated with 4-days paraquat, 1-day caffeine, or 4-days quinine, compared to the non-treated control group. Three independent experiments were performed for each group, P>0.05, One-way ANOVA. C. The level of *Dcp2* mRNA was significantly suppressed by 4 days of nicotine treatment in *w^1118^* flies (*P<0.05) but not in *Dcp2^V1194^* and *Dcp2^c02419^* flies (N.S., P>0.05). Three independent experiments were performed for each group, *t*-test. D–E. cNILH was blocked in *elav*-GAL4>UAS-*Dcp2* (D) and *elav*-GAL4>UAS-*Dcp1* ^RNAi^ (E) flies (N.S., P>0.05), while their parental control flies developed cNILH (*P<0.05) (Mann-Whitney U test). These flies showed normal basal locomotor activity when compared to their parental control groups (N.S., P>0.05, one-way ANOVA). n = 28–32 for each group. F. Levels of *Dcp1* mRNA in the fly head were not affected by 4 days of nicotine treatment (Three independent experiments were performed for each group, N.S., P>0.05, *t*-test). N.S. indicates no significant difference. Bars and error bars represent the mean ± SEM.

We then asked whether *Dcp2* expression is regulated by nicotine exposure in wild-type flies. Using a relative qPCR assay, we examined the *Dcp2* mRNA level in the heads of *w^1118^* flies treated with nicotine for 0–5 day(s). Compared to the null treatment (0-day) group, *Dcp2* mRNA was slightly elevated in the 1-day treatment group. In the 3-, 4-, and 5-day treatment groups (Fig. 5A), the level of *Dcp2* mRNA was significantly suppressed to approximately 50% of the null treatment group. The dynamic change in *Dcp2* mRNA levels suggests that Dcp2 plays an essential role in responding to and mediating nicotine signals. Notably, *Dcp2* mRNA is down-regulated after 3 days of exposure to nicotine-containing food, one day before cNILH occurred.

In view of the neurotoxicity of nicotine, we asked whether the down-regulation of *Dcp2* mRNA was a general response to environmental stress. Using the same nicotine treatment paradigm, we treated wild type flies with paraquat (an agent used to induce oxidative-stress) and found that the level of *Dcp2* mRNA in these flies was not different from untreated controls (Fig. 5B). As shown above (Fig. 2C–E), one day of caffeine treatment can elevate total locomotor activity in adult flies. However, caffeine did not significantly alter *Dcp2* mRNA levels after the first day of treatment (Fig. 5B). We then tested if reduced food intake could affect *Dcp2* mRNA level and found that the level did not change when flies were treated with 2.0 mM quinine-containing food for 4 days (Fig. 5B). Taken together, these results suggest that chronic nicotine exposure triggers a Dcp2-dependent signal pathway, which is not activated by general stress or other drug stimuli.

Next, we tested whether nicotine could suppress *Dcp2* expression in *Dcp2* mutant flies. After 4 days of nicotine treatment, the level of *Dcp2* mRNA was not decreased in *Dcp2^V1194^* flies compared to untreated control flies (Fig. 5C). Although *Dcp2^c02419^* flies have comparable *Dcp2* mRNA levels to control flies, *Dcp2* mRNA is not down-regulated by chronic nicotine administration (Fig. 5C), supporting our hypothesis that the insertion in the 3′UTR of *Dcp2* disrupts its regulation. Taken together, we suggest that down-regulation of Dcp2 is required for the development of cNILH.

The next question we asked was whether we could block cNILH by blocking the down-regulation of *Dcp2* mRNA via overexpressing it. We generated a UAS-*Dcp2* fly line by cloning full length *Dcp2* cDNA into the pUAST vector, and found that the level of *Dcp2* mRNA increased about 4 times in *elav*-GAL4>UAS-*Dcp2* flies compared to the parental controls (Fig. S6B). These flies exhibited comparable basal locomotor hyperactivity; however, they failed to develop the cNILH observed in their parental control (Fig. 5D). These results demonstrate that excessive Dcp2 also prevents flies from developing nicotine-induced locomotor hyperactivity, suggesting that cNILH requires the suppression of Dcp2.

To further investigate whether the decapping function of Dcp2 is required for mediating cNILH, we examined another major component of the decapping complex, Dcp1, using a UAS-*Dcp1* ^RNAi^ fly line (V31441). Similar to *Dcp2*-silenced flies, cNILH was also blocked in *elav*-GAL4>UAS-*Dcp1* ^RNAi^ flies (Fig. 5E), in which *Dcp1* expression was knocked-down to half of its normal level in the nervous system (Fig. S6C). However, in contrast to *Dcp2*-deficient flies, the basal locomotor activity of Dcp1 knock-down flies was comparable to their parental controls, suggesting that Dcp2 has a more important function than Dcp1 in the cNILH response. Moreover, *Dcp1* mRNA levels in *w^1118^* flies were not affected by nicotine administration (Fig. 5F), suggesting that nicotine modulates decapping signaling by specifically targeting Dcp2.

How is the level of *Dcp2* mRNA down-regulated by nicotine-triggered signals? Although Dcp2 is a key factor in mRNA degradation, there are no reports to date on its regulation. Generally speaking, *Dcp2* expression may be regulated transcriptionally or post-transcriptionally. Results from *Dcp2* ^c02419^ mutant flies suggest that microRNA pathway-mediated post-transcriptional regulation may play an essential role in nicotine-induced down-regulation of *Dcp2* mRNA. In this fly line, there is an insertion in the 3′UTR region of *Dcp2* that does not alter the basal *Dcp2* mRNA level but does block the down-regulation of *Dcp2* mRNA, as well as locomotor hyperactivity, upon nicotine administration. The 3′UTR is the microRNA targeting region, and the microRNA pathway negatively regulates mRNA stability and translation [48]. A rodent microRNA microarray study in a rat PC12 cell model has shown that nicotine can selectively modulate the expression of multiple microRNAs, demonstrating that the microRNA pathway is one of the molecular mechanisms involved in the nicotine-triggered regulation of gene expression [49]. We assayed the 3′UTR of *Drosophila Dcp2* with some online prediction tools, and several binding sites (including miR-277 and miR-375) were identified. Thus, we propose that in response to microRNA fluctuations caused by nicotine treatment, *Dcp2* expression is modified, leading to more profound and long-lasting effects. The mechanism of *Dcp2* regulation upon nicotine exposure needs to be systematically investigated in the future.

### Conclusion

In summary, we demonstrated that continuous nicotine exposure induced both locomotor hyperactivity and *Dcp2* mRNA down-regulation in wild-type adult flies, while flies with either a low level of *Dcp2* mRNA (*Dcp2^V1194^*, *Dcp2^V1194/e00034^*, and *elav*-GAL4>UAS-*Dcp2* ^RNAi^ flies, group 1) or insufficient suppression of *Dcp2* mRNA (*Dcp2^c02419^* and *elav*-GAL4>UAS-*Dcp2* flies, group 2) failed to develop chronic nicotine-exposure-induced locomotor hyperactivity. Group 1 flies exhibited high basal locomotor activity without nicotine treatment, while group 2 flies showed normal basal locomotor activity. Thus, a low level of *Dcp2* mRNA is sufficient to promote locomotor hyperactivity without nicotine, while sufficient suppression of *Dcp2* by nicotine is required for developing locomotor hyperactivity. We also suggested that the microRNA pathway is involved in nicotine-signal-mediated Dcp2 regulation and that the mechanism of their interaction deserves more investigation in future research. Our findings reveal a significant role for the mRNA decapping pathway in developing locomotor hyperactivity in response to chronic nicotine exposure and hint that the regulation of mRNA degradation is involved in the development of nicotine dependence.

## Materials and Methods

### Fly Stocks

Flies were reared on the Bloomington *Drosophila* Stock Center standard medium at 25°C and 60% humidity under a 12/12 light/dark cycle unless indicated otherwise. Canton-S (CS) and *w^1118^* (from VDRC) flies were used as the wild-type and genetic background flies, respectively. The following RNAi fly lines were obtained from the Vienna *Drosophila* RNAi Center [44] (stock numbers are listed): UAS-*nAChR-α96Ab* ^RNAi^ (V1194 and V1195); UAS-*nAChR-α96Aa* ^RNAi^ (V1189); UAS-*nAChR-gfa* ^RNAi^ (V11329); UAS-*nAChR-β21C* ^RNAi^ (V42740); UAS-*nAChR-β64B* ^RNAi^ (V33824); UAS-*nAChR-α80B* ^RNAi^ (V12441); UAS-*nAChR-β96A* ^RNAi^ (V1200); UAS-*nAChR-α30D* ^RNAi^ (V8890 and V8889); UAS-*Dcp1* ^RNAi^ (V31441), and UAS-*Dcp2* ^RNAi^ (V22272). Another UAS-*Dcp2* ^RNAi^ line (TH0652) was obtained from the Tsinghua Fly Center, a resource of *Drosophila* transgenic RNAi lines constructed using the VALIUM vector [50]. The V22272 line targets to 363 bp (see VDRC website) in the exon 3 region of *Dcp2*, and the TH0652 line targets 21 bp in the exon 1 region of *Dcp2*. The *elav*-GAL4 (B25750, B23868) and *Dcp2*-GAL4 (*Dcp2^GAL4^*, BG01766) lines were obtained from the Bloomington Stock Center. The UAS-mCD8::GFP line was used for immunofluorescence imaging. The *Dcp2* mutant flies *Dcp2^e00034^* and *Dcp2^c02419^* were obtained from the Harvard Exelixis Stock Center. To keep the genetic background consistent, all of the *Dcp2* mutants and RNAi fly lines were outcrossed to *w^1118^* flies for 5 generations.

UAS*-Dcp2* flies were generated by cloning the full length *Dcp2* cDNA into the pUAST vector. The forward primer was AGATCTATGGAGCTAAACAATCTAATACGTA, and the reverse primer was GGTACCGCAAAACACATTTGCTATGAAGT. Microinjection was performed by Rainbow Transgenic Flies, Inc. U.S.A., and three individual transgenic lines were maintained.

### Drug Treatment

The drugs and reagents used in this study included nicotine (Sigma), caffeine (Sigma-Aldrich), paraquat (Sigma-Aldrich), and mecamylamine (Sigma). One or two of these compounds (as specified in the text) were mixed into standard fly food during food preparation and were stored in a refrigerator at 4°C for up to one week.

Male flies were collected within 1 day after eclosion, grouped at 50 per vial, and starved for 2 h before drug treatment. For the standard 4-day nicotine treatment, flies were raised on food containing 0.6, 1.8, 3.0, or 4.2 mM nicotine for 3 days in the vials, and then, on the 4^th^ day, the flies were individually transferred to monitor tubes containing food with the same dose of nicotine for locomotor activity recording. Any divergence from this procedure is specified in the text. Paraquat treatment was delivered via the food at a dose of 1 mg/mL for three days, in line with a previous report [51]. For caffeine treatment, flies were transferred and monitored on 2.5 mg/mL of caffeine-containing food for 1 day [45]. During the feeding and/or recording periods, 0.75 mM mecamylamine was added to the 3 mM nicotine-containing food to block nAChRs.

### Locomotor Activity Assay

Flies were individually introduced into the *Drosophila* Activity Monitoring System (TriKinetics, USA) and were monitored for 1 day by infrared rays [52]. Regular or conditional food with drugs was provided. Wake time was defined as at least 1 movement detected within 5 min (≥1 move count/5 min), and Total Wake Time was the sum of the wake time. The total locomotor activity (TLA) in one day was defined as the sum of the movement counts during a 24-hour period.

### Inverse PCR (iPCR)

iPCR assays were performed to identify the insertion site of the P-element in the V1194 flies according to a standard protocol from the Berkeley *Drosophila* Genome Project. Briefly, genomic DNA was extracted from approximately 30 flies, then purified (QIAGEN DNeasy kit), digested by Sau3A I (NEB) for 8 hr, and self-ligated (T4 DNA Ligase, NEB) for 2 hr at 25°C. The product was used as the template for PCR with the P-31: CGACGGGACCACCTTATGTTATTTCATCATG, pWiz-F1: TAGAGCCAGATATGCGAGCAC, and pWiz-R1: GTCCGTGGGGTTTGAATTAAC primers. The PCR products were purified, sequenced and aligned to the *Drosophila* genomic sequence using BLAST.

### Real-time Quantitative PCR (qPCR)

RNA was extracted from 30–50 whole flies or approximately 200 fly heads with TRIzol (Invitrogen). RNA quality was assessed using the Lab-on-a-Chip 2100 Bioanalyzer (Agilent) platform. Two micrograms of total RNA was treated with RQ1 DNase (Promega) and then reverse transcribed using oligo(dT) primers and Superscript III reverse transcriptases (Invitrogen). Real-time PCR was performed with a SYBR Premix Ex Taq™ II kit (Takara) using an ABI PRISM 7000 real-time PCR Detection system (Applied Biosystems). The relative mRNA level was calculated using the comparative *C_T_* method [53]. *rp49* was used as the reference gene. Three repeats were performed for each sample, and data were collected and analyzed from 3 independent samples.

### Primer Sequences Used for qPCR

*Dcp2*: 5′-AAGCGTCAACTGTTCCATAGCC-3′, 5′-TGCGCCTTAGCTGCCTTAAGT-3′;

*Dcp1*: 5′-GTCCAGGCCTTCACGTACCTTA-3′, 5′-TGATATGTGGAGCTAGAGTCCA-3′;

*rp49*: 5′-CCAAGGACTTCATCCGCCACC-3′, 5′-GCGGGTGCGCTTGTTCGATCC-3′;

*dbo*: 5′-GGAGCGCTACGATCCAAAAGA-3′, 5′-CCGCCAATTGCGTAGAGAAA-3′;

*nAChR-α96Ab*: 5′-FAAACTCCTGCTGATGCGTGTG-3′, 5′-GCCCGAGTTCATTTGCATCTC-3′.

### Feeding Assay

The feeding assay was modified from previously reported methods [38], [54]. Briefly, male flies were collected within 1 day after eclosion and were reared at 25°C on normal food or on nicotine- or quinine-containing food. A group of 100 flies were starved for 12 hr and transferred to blue-colored food (1.25 mg/ml, FD&C Blue No.1, McCormick) for 2 hr. After feeding, the flies were frozen and their bodies were separated from their heads and homogenized in PBS buffer (PBS, 1.86 mM NaH2PO4, 8.41 mM Na2HPO4, 175 mM NaCl). After centrifuging, the supernatant was measured for absorbance at 625 nm. The absorbance value for flies fed with blue food was subtracted from that for flies fed with normal food. The net absorbance value reflected the food intake. The feeding profile for each line was determined by 5–6 independent experiments.

### Nicotine Concentration Assay

Flies were fed with normal food or 3 mM nicotine-containing food for 4 consective days, and nicotine concentration was measured by HPLC at the end of 4^th^ day [55]. Each sample included about 200 whole flies or 1,000 heads, and every group included three independent samples. The samples were homogenized in 10 ml of extraction buffer containing 10 g/L trichloroacetic acid (Sigma-Aldrich) and 0.44 g/L lead acetate trihydrate (Sigma-Aldrich). The tubes were vigorously shaken and placed in an ultrasonic bath for 30 min, then centrifuged at 10,000 rpm for 10 min. The extraction was repeated three times. The supernatant fluid was transferred to a new tube for the HPLC nicotine assay. The nicotine concentration of the supernatant fluid was assayed by Sino Analytica Company, China and normalized to the body weight.

### Immunofluorescence

Adult male flies (4 days after eclosion at 25°C) were collected and brains were dissected in ice-cold PBS then fixed in 4% paraformaldehyde (PFA) solution in PBS for 1 hr at room temperature. After three 20 min washings in PBS containing 0.3% Triton X-100 (PBT), the brains were blocked with 5% normal goat serum (NGS, Invitrogen, 01-6201) in PBT for 1 hr. The brains were then incubated with primary antibodies in blocking solution at 4°C for 48 hr. After six 20 min washings in PBT, the samples were incubated with secondary antibodies at 4°C for 12 hr. The primary antibodies used were mouse anti-nc82 (1∶50, DSHB) and rabbit anti-GFP (1∶100, Sigma, A6455). The secondary antibodies (1∶500, Molecular Probes) used were Alexa 633 goat anti-mouse IgG (A21053) and Alexa 488 goat anti-rabbit IgG (A11008). Brains were finally washed six times with PBT and then mounted in Vectashield (Vector Laboratories, California, USA). Confocal microscopy was performed on a Nikon FN1 confocal microscope and the images were analyzed with Image J (US National Institute of Health).

### Statistical Analysis

All statistical analyses were conducted using Sigmastat. The Wilks-Shapiro test was used to determine normality of the data. Normally distributed data were analyzed using two-tailed, unpaired Student’s *t*-tests or one or two-way ANOVA, followed by the Tukey-Kramer HSD Test as the post hoc test. Non-parametrically distributed data were assessed using the Mann-Whitney U test. Data are presented as mean values, and error bars represent the standard error of the mean (SEM). Differences between the groups were considered significant if the probability of error was less than 0.05 (P<0.05).

## Supporting Information

Figure S1 **Continuous nicotine administration is required for developing locomotor hyperactivity.** CS male flies were collected within 1 day after hatching and were divided into four groups: the 4-day nicotine-containing food group, the 3-day nicotine-containing and 1-day normal food group, the 3-day normal and 1-day nicotine-containing food group, and the 4-day normal food group (control). The locomotor activity of flies was recorded on the 4^th^ day. Continuous nicotine treatment for 4 days induced cNILH at all doses tested (0.6, 1.8 and 3.0 mM) when compared to control flies (***P<0.001), while the other two groups showed no significant differences in total locomotor activity compared to the control group (P>0.05). n = 28–32, one-way ANOVA. Bars and error bars represent the mean ± SEM.
(TIF)

Figure S2 **Chronic nicotine intake-induced locomotor hyperactivity is not due to decreased food intake.** A. Wild-type CS flies fed with blue food in the food consumption experiment. B. Histogram of food consumption in CS flies fed with control food or 3 mM nicotine-containing food. N-0 to N-4: Before the food consumption test, five groups of flies were treated with nicotine-containing food for 0 to 4 days. Food consumption was reduced by approximately 50% in N-0 naive flies compared to the control group (***P<0.001) and recovered to approximately 66–84% in N-1 to N-4 flies (*P<0.05). 5–6 independent experiments, one-way ANOVA. C. Histogram of food consumption in CS flies fed with control food or 2.0 mM quinine-containing food. Q-0 to Q-4: five groups of flies were pre-fed with quinine-containing food for 0 to 4 days. Similar to the nicotine groups, food consumption was significantly reduced in the Q-0 group (***P<0.001) and was recovered in the other groups (*P<0.05). 5–6 independent experiments, one-way ANOVA. D. There were no significant differences in the total locomotor activity counts over a 24 h period between Q-1 to Q-4 flies and control flies (n = 28–32, N.S. indicates no significant difference, P>0.05, one-way ANOVA). E. Locomotor activity curves quantified every 30 min for 24 hr in flies treated with quinine-containing and control food. Bars and error bars represent the mean ± SEM.
(TIF)

Figure S3 **Two nAChR-subunits are required for developing cNILH.** Using the 4-day nicotine treatment and recording paradigm, nicotine receptor subunits were tested for their role in the cNILH effect via an RNAi approach. Two *elav*-GAL4 lines (on X or III chromosome) were used to drive the expression of UAS-*nAChR-subunit* ^RNAi^ in the nervous system. Among the eight nAChR subunits (the ten lines used are labeled by their VDRC stock numbers) tested, cNILH was only blocked in flies in which *nAChR*-*α96Ab* or -*gfa* was knocked down (N.S. indicates no significant difference, P>0.05). Flies in which other subunits were knocked down and flies from all parental control groups exhibited normal cNILH (***P<0.001). n = 28–32, Mann-Whitney U test. Bars and error bars represent the mean ± SEM.
(TIF)

Figure S4 **Long-term nicotine administration induces locomotor hyperactivity in** ***w^1118^*** **and V1195 flies, but not in V1194 flies.** A–B. Male flies were collected within 1 day after hatching and were transferred to activity monitor tubes on the 4^th^ day. The groups were switched from normal food to nicotine-containing food on different days. The group number indicates how many consecutive days the flies were treated with nicotine-containing food. Locomotor activity was recorded on the 4^th^ day in groups 1–4, while group 5 was monitored on the 5^th^ day. The total locomotor activity (TLA) was normalized to the non-treatment control group for each day. A. Compared to the non-treatment control, nicotine-containing food at dosages of 1.8, 3.0, and 4.2 mM induced locomotor hyperactivity on the 4^th^ day in *w^1118^* flies (n = 28–32, **P<0.01, ***P<0.001, one-way ANOVA), but a 0.6 mM dose did not. On the 5^th^ day, cNILH was unstable across the different experiments and dosages. B. V1194 flies failed to develop locomotor hyperactivity at any nicotine dose over 5 days, and TLA showed a significant decrease at most nicotine doses. (n = 28–32, **P<0.01, ***P<0.001, one-way ANOVA), C. Chronic nicotine-induced locomotor hyperactivity in V1195 flies (***P<0.001). The basal locomotor activity of V1194 flies was significantly higher than that of V1195 flies (**P<0.01). n = 32, *t*-test. Bars and error bars represent the mean ± SEM.
(TIF)

Figure S5 **Caffeine induces locomotor hyperactivity in all** ***Dcp2*** **mutant, knock-down, and overexpressing flies, as in control flies.** Male flies were collected within 1 day after hatching and kept on normal food for 3 days. On the 4^th^ day, flies were transferred to individual activity monitor tubes with caffeine-containing or control food to record their locomotor activity for one day. Total locomotor activity (TLA) per fly group was significantly elevated when flies were treated with caffeine-containing food compared with the control food group (n = 28–32, ***P<0.001, Mann-Whitney U test). Bars and error bars represent the mean ± SEM.
(TIF)

Figure S6 **RNAi and overexpression efficiency assays for** ***Dcp2*** **and** ***Dcp1*** **.** Total RNA was extracted from approximately 100 heads of male flies for each group. The mRNA levels of tested genes were normalized to *rp49* mRNA. A. Compared to parental controls, *Dcp2* mRNA levels were down-regulated significantly by approximately 50% in *elav*-GAL4>UAS-*Dcp2* ^RNAi−V22272^ and 20% in *elav*-GAL4>UAS-*Dcp2* ^RNAi−TH0652^ flies (*P<0.05, **P<0.01, ***P<0.001). B. Compared to parental controls, the level of *Dcp2* mRNA was significantly increased (approximately 4-fold) in *elav*-GAL4>UAS-*Dcp2* flies (***P<0.001). C. Compared to parental controls, the level of *Dcp1* mRNA was significantly reduced to approximately 50% in *elav*-GAL4>UAS-*Dcp1* ^RNAi^ flies (**P<0.01). Three independent experiments were performed for each group, one-way ANOVA. Bars and error bars represent the mean ± SEM.
(TIF)
