# Supplementary figures and images for: Down-Regulation of Decapping Protein 2 Mediates Chronic Nicotine Exposure-Induced Locomotor Hyperactivity in Drosophila
Source: PLoS One. 2012 Dec 26;7(12):e52521. doi: 10.1371/journal.pone.0052521 (PMC3530533; doi:10.1371/journal.pone.0052521)

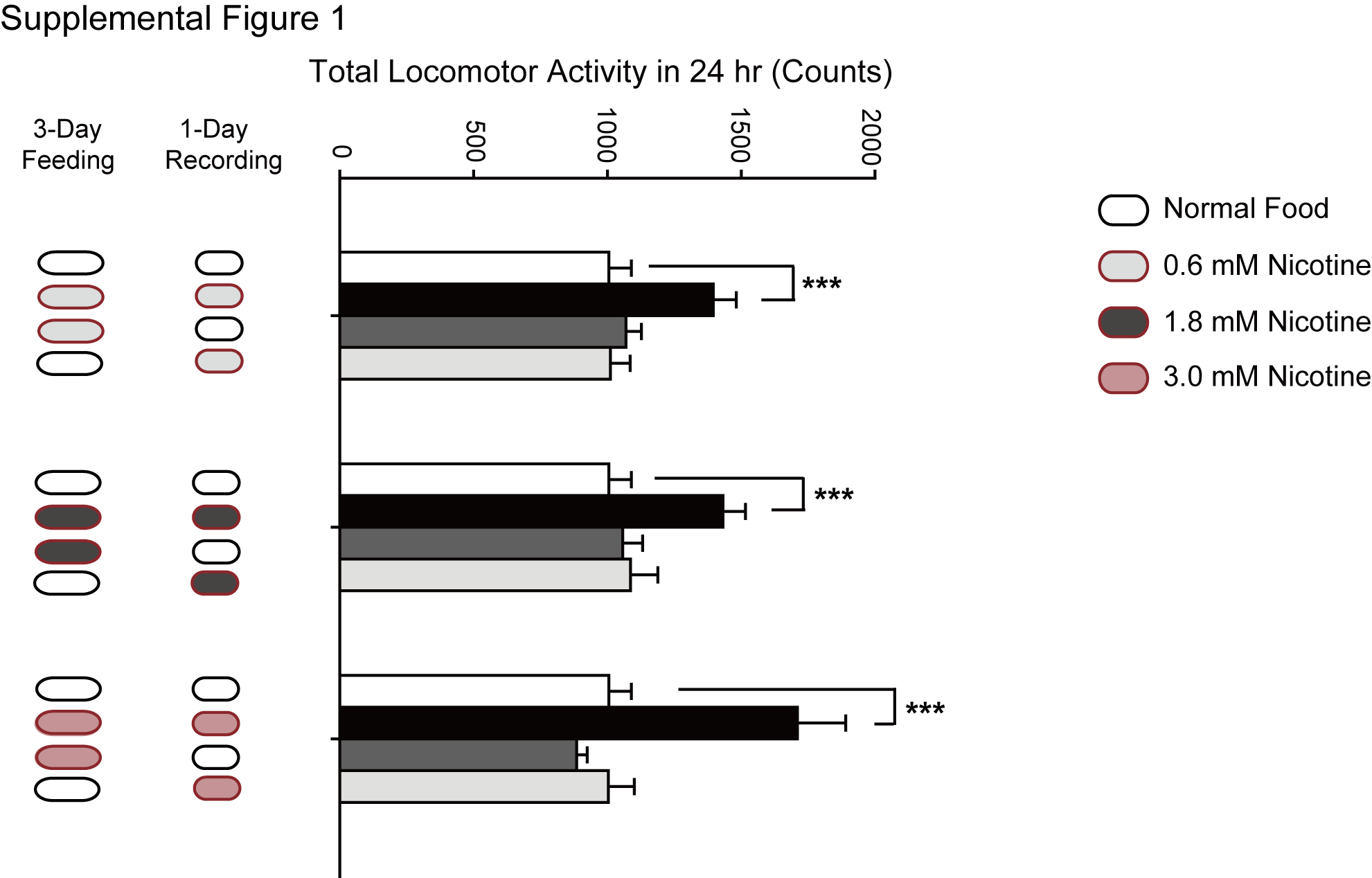

Supplement: Figure S1 — Continuous nicotine administration is required for developing locomotor hyperactivity. CS male flies were collected within 1 day after hatching and were divided into four groups: the 4-day nicotine-containing food group, the 3-day nicotine-containing and 1-day normal food group, the 3-day normal and 1-day nicotine-containing food group, and the 4-day normal food group (control). The locomotor activity of flies was recorded on the 4th day. Continuous nicotine treatment for 4 days induced cNILH at all doses tested (0.6, 1.8 and 3.0 mM) when compared to control flies (***P<0.001), while the other two groups showed no significant differences in total locomotor activity compared to the control group (P>0.05). n = 28–32, one-way ANOVA. Bars and error bars represent the mean ± SEM. (TIF) [file pone.0052521.s001.tif]

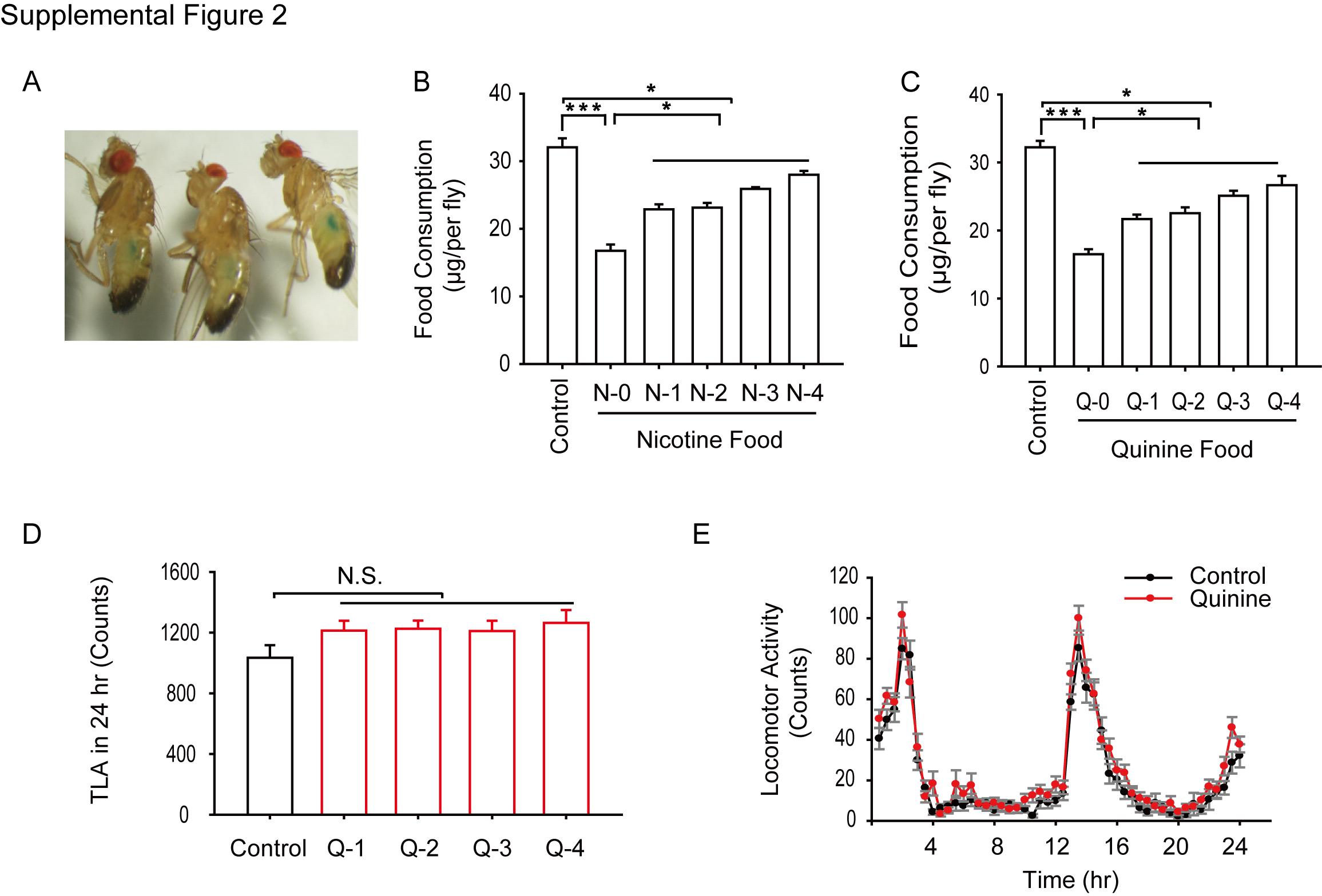

Supplement: Figure S2 — Chronic nicotine intake-induced locomotor hyperactivity is not due to decreased food intake. A. Wild-type CS flies fed with blue food in the food consumption experiment. B. Histogram of food consumption in CS flies fed with control food or 3 mM nicotine-containing food. N-0 to N-4: Before the food consumption test, five groups of flies were treated with nicotine-containing food for 0 to 4 days. Food consumption was reduced by approximately 50% in N-0 naive flies compared to the control group (***P<0.001) and recovered to approximately 66–84% in N-1 to N-4 flies (*P<0.05). 5–6 independent experiments, one-way ANOVA. C. Histogram of food consumption in CS flies fed with control food or 2.0 mM quinine-containing food. Q-0 to Q-4: five groups of flies were pre-fed with quinine-containing food for 0 to 4 days. Similar to the nicotine groups, food consumption was significantly reduced in the Q-0 group (***P<0.001) and was recovered in the other groups (*P<0.05). 5–6 independent experiments, one-way ANOVA. D. There were no significant differences in the total locomotor activity counts over a 24 h period between Q-1 to Q-4 flies and control flies (n = 28–32, N.S. indicates no significant difference, P>0.05, one-way ANOVA). E. Locomotor activity curves quantified every 30 min for 24 hr in flies treated with quinine-containing and control food. Bars and error bars represent the mean ± SEM. (TIF) [file pone.0052521.s002.tif]

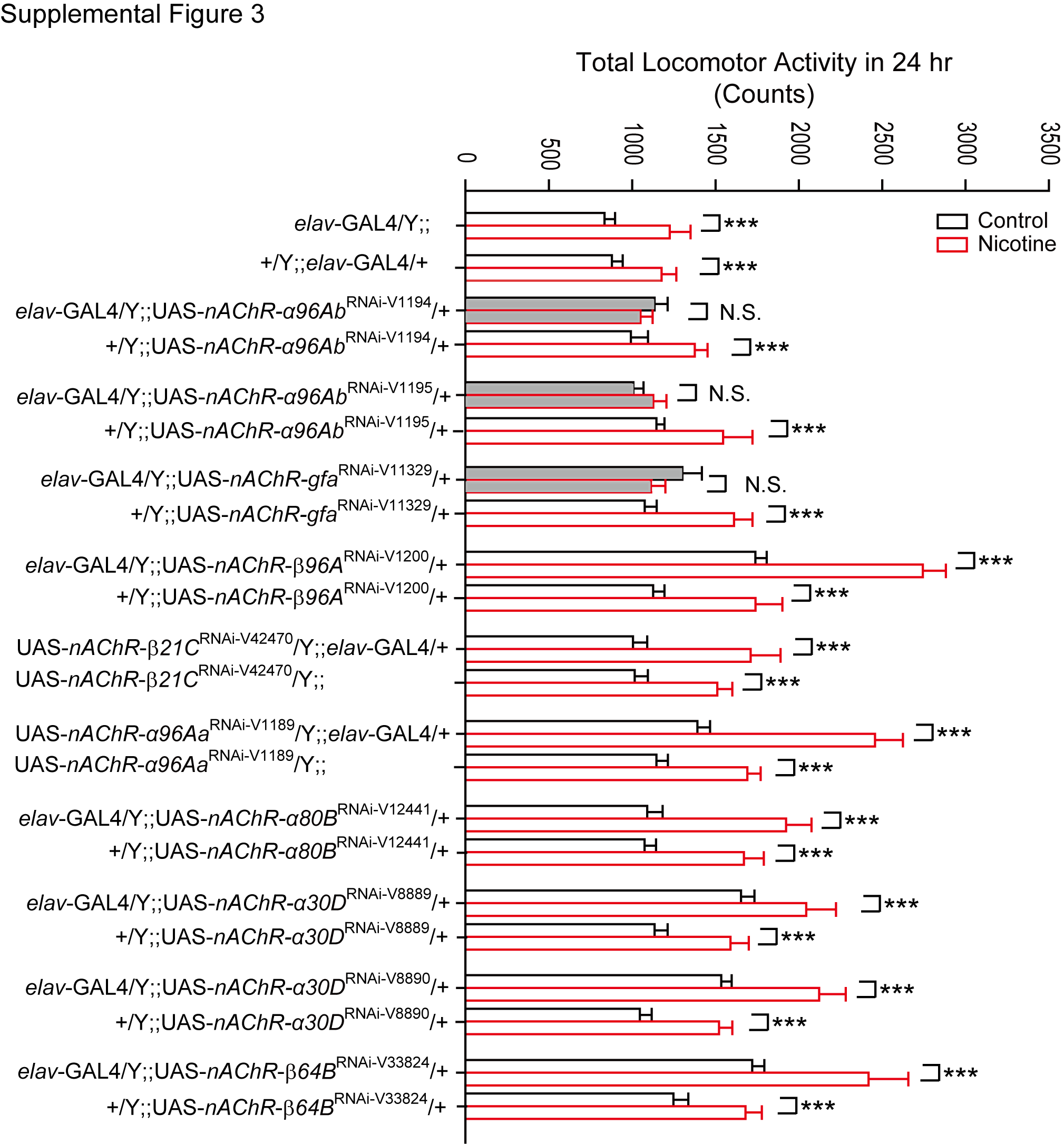

Supplement: Figure S3 — Two nAChR-subunits are required for developing cNILH. Using the 4-day nicotine treatment and recording paradigm, nicotine receptor subunits were tested for their role in the cNILH effect via an RNAi approach. Two elav-GAL4 lines (on X or III chromosome) were used to drive the expression of UAS-nAChR-subunit RNAi in the nervous system. Among the eight nAChR subunits (the ten lines used are labeled by their VDRC stock numbers) tested, cNILH was only blocked in flies in which nAChR-α96Ab or -gfa was knocked down (N.S. indicates no significant difference, P>0.05). Flies in which other subunits were knocked down and flies from all parental control groups exhibited normal cNILH (***P<0.001). n = 28–32, Mann-Whitney U test. Bars and error bars represent the mean ± SEM. (TIF) [file pone.0052521.s003.tif]

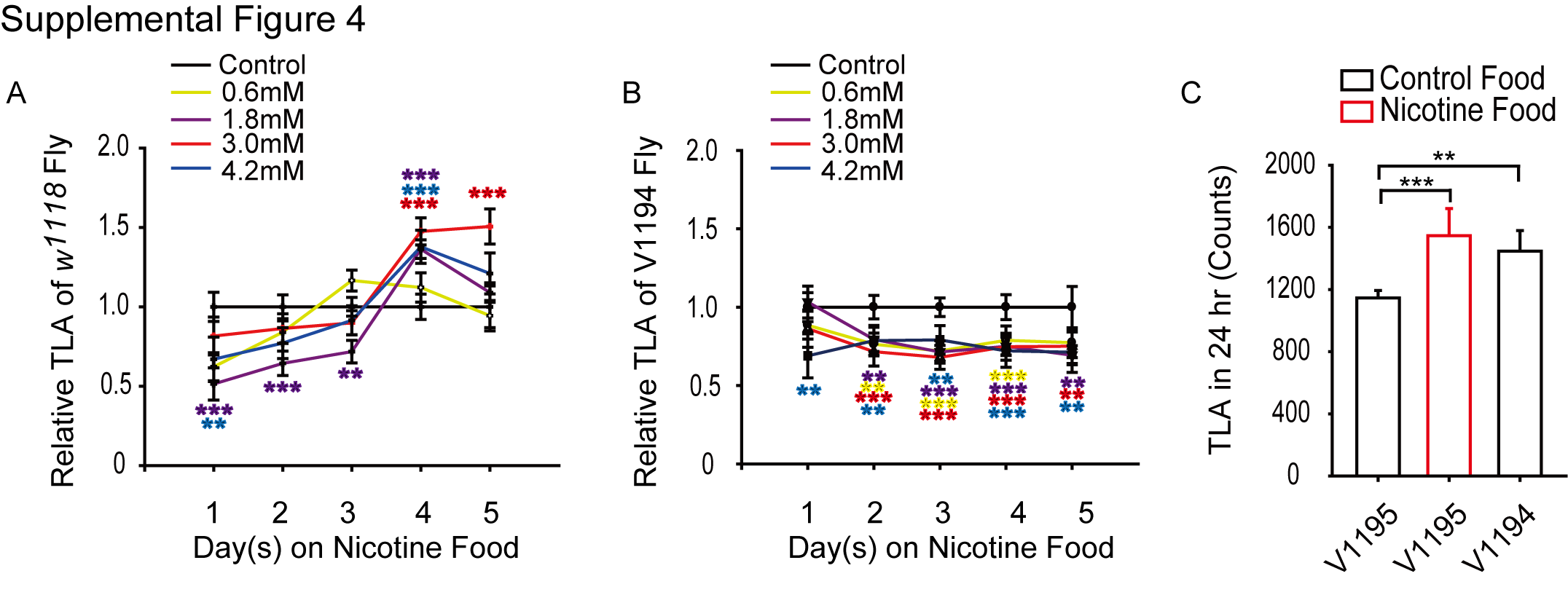

Supplement: Figure S4 — Long-term nicotine administration induces locomotor hyperactivity in w1118 and V1195 flies, but not in V1194 flies. A–B. Male flies were collected within 1 day after hatching and were transferred to activity monitor tubes on the 4th day. The groups were switched from normal food to nicotine-containing food on different days. The group number indicates how many consecutive days the flies were treated with nicotine-containing food. Locomotor activity was recorded on the 4th day in groups 1–4, while group 5 was monitored on the 5th day. The total locomotor activity (TLA) was normalized to the non-treatment control group for each day. A. Compared to the non-treatment control, nicotine-containing food at dosages of 1.8, 3.0, and 4.2 mM induced locomotor hyperactivity on the 4th day in w1118 flies (n = 28–32, **P<0.01, ***P<0.001, one-way ANOVA), but a 0.6 mM dose did not. On the 5th day, cNILH was unstable across the different experiments and dosages. B. V1194 flies failed to develop locomotor hyperactivity at any nicotine dose over 5 days, and TLA showed a significant decrease at most nicotine doses. (n = 28–32, **P<0.01, ***P<0.001, one-way ANOVA), C. Chronic nicotine-induced locomotor hyperactivity in V1195 flies (***P<0.001). The basal locomotor activity of V1194 flies was significantly higher than that of V1195 flies (**P<0.01). n = 32, t-test. Bars and error bars represent the mean ± SEM. (TIF) [file pone.0052521.s004.tif]

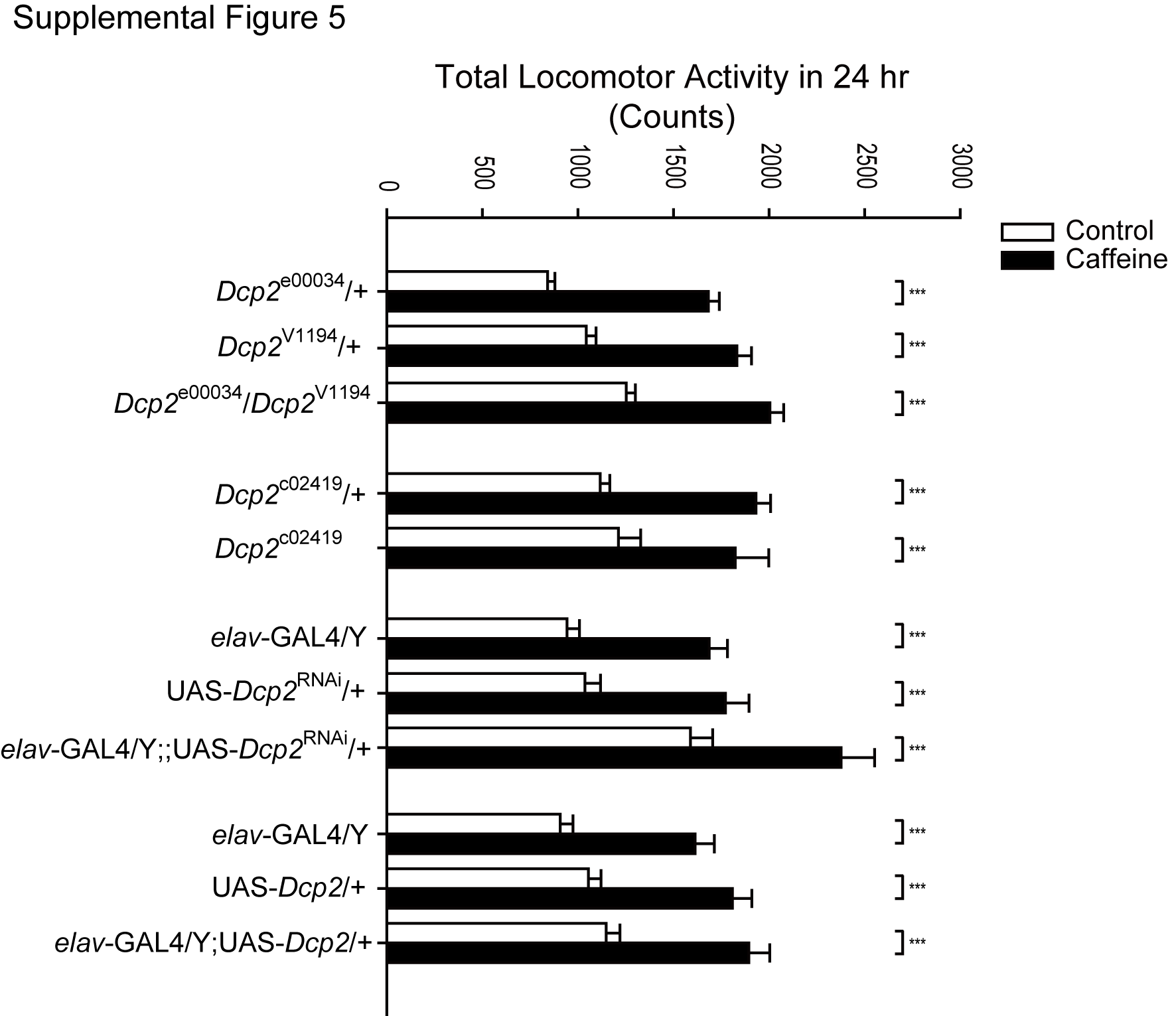

Supplement: Figure S5 — Caffeine induces locomotor hyperactivity in all Dcp2 mutant, knock-down, and overexpressing flies, as in control flies. Male flies were collected within 1 day after hatching and kept on normal food for 3 days. On the 4th day, flies were transferred to individual activity monitor tubes with caffeine-containing or control food to record their locomotor activity for one day. Total locomotor activity (TLA) per fly group was significantly elevated when flies were treated with caffeine-containing food compared with the control food group (n = 28–32, ***P<0.001, Mann-Whitney U test). Bars and error bars represent the mean ± SEM. (TIF) [file pone.0052521.s005.tif]

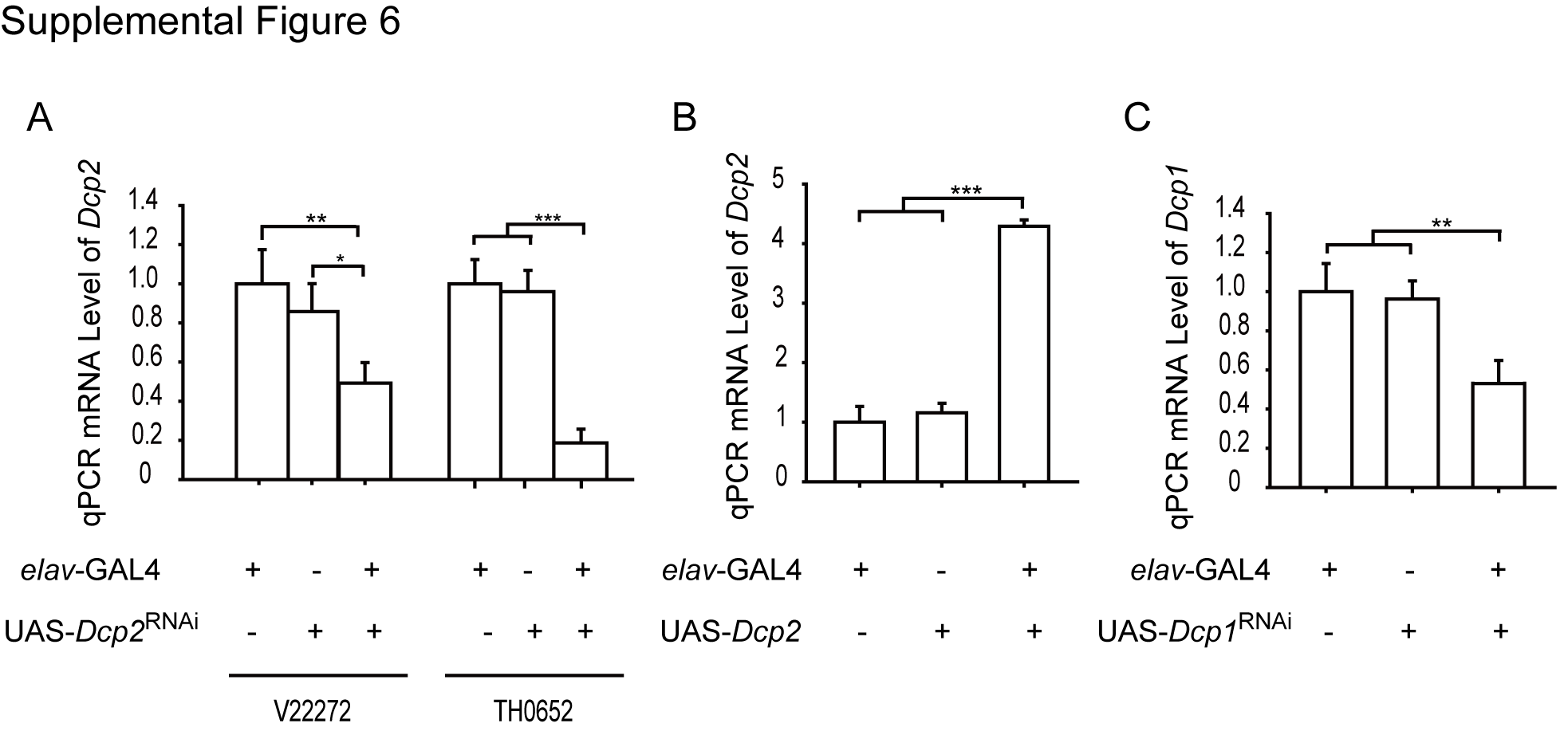

Supplement: Figure S6 — RNAi and overexpression efficiency assays for Dcp2 and Dcp1 . Total RNA was extracted from approximately 100 heads of male flies for each group. The mRNA levels of tested genes were normalized to rp49 mRNA. A. Compared to parental controls, Dcp2 mRNA levels were down-regulated significantly by approximately 50% in elav-GAL4>UAS-Dcp2 RNAi−V22272 and 20% in elav-GAL4>UAS-Dcp2 RNAi−TH0652 flies (*P<0.05, **P<0.01, ***P<0.001). B. Compared to parental controls, the level of Dcp2 mRNA was significantly increased (approximately 4-fold) in elav-GAL4>UAS-Dcp2 flies (***P<0.001). C. Compared to parental controls, the level of Dcp1 mRNA was significantly reduced to approximately 50% in elav-GAL4>UAS-Dcp1 RNAi flies (**P<0.01). Three independent experiments were performed for each group, one-way ANOVA. Bars and error bars represent the mean ± SEM. (TIF) [file pone.0052521.s006.tif]
